# Cross-Cultural Adaptation and Clinimetric Testing of Functional Rating Index (FRI) Outcome Measure into the Arabic Language

**DOI:** 10.1155/2022/6229847

**Published:** 2022-06-23

**Authors:** Saad M. Alsaadi, Raafat Ahmed, Kawther Alotaibi, Matar Abdullah Alzahrani, Nasser Alotaibi, Fayez M. Alahmri, Arun Vijay Subbarayalu

**Affiliations:** ^1^Physiotherapy Department, College of Applied Medical Sciences, Imam Abdulrahman Bin Faisal University, Physiotherapy Department, King Fahd Hospital of the University, Khobar, Saudi Arabia; ^2^Physical Therapy Department, College of Applied Medical Sciences, Imam Abdulrahman Bin Faisal University, P.O. Box 1982, Dammam 31441, Saudi Arabia; ^3^BANA Physical Therapy Center, P.O. Box 34257, Dhahran 8350, Saudi Arabia; ^4^Department of Physiotherapy, King Fahd Hospital of the University, Imam Abdulrahman Bin Faisal University, P.O. Box 40244, Khobar 31952, Saudi Arabia; ^5^Department of Medical Rehabilitation, Ministry of Health, Riyadh, Saudi Arabia; ^6^Physiotherapy Department, Quality Assurance Department, Deanship of Quality and Academic Accreditation, Imam Abdulrahman Bin Faisal University, P.O. Box 1982, Dammam 31441, Saudi Arabia

## Abstract

**Background:**

The Functional Rating Index (FRI) is a self-report scale widely used to determine the level of disability in low back pain (LBP) populations.

**Objectives:**

This study was aimed at conducting the cross-cultural adaptation of the FRI-Arabic version (FRI-Ar) and testing the clinometric properties of FRI-Ar.

**Methods:**

The cross-cultural adaptation process was used to develop the FRI-Ar. This study included acute and subacute LBP patients. Each patient was asked to complete the questionnaires at three time points: baseline, 24-hour follow-up, and two-week follow-up. The questionnaires used were FRI-Ar, Roland-Morris Disability Questionnaire (RMDQ), Oswestry Disability Index (ODI), Numerical Pain Rating Scale (NPRS), Global Perceived Effect Scale (GPE), and Patient-Specific Functional Scale (PSFS). Statistical analysis was carried out to measure the instrument's reliability, validity, and responsiveness.

**Results:**

The FRI was cross-culturally adapted to the Arabic language, and the adapted version was validated. Two hundred patients completed the questionnaires at the baseline; however, 120 patients completed the questionnaires at 24-hour and two-week follow-up. Cronbach's alpha, interclass correlation coefficient (ICC_2,1_), standard error of measurement (SEM), and minimal detectable change (MDC_95%_) for the FRI-Ar were observed as 0.85, 0.85, 1.17 (2.9%), and 3.24, respectively. The FRI-Ar showed a moderate positive correlation only with the RMDQ, ODI, and NPRS (*p* < 0.05). Also, it showed the responsiveness with a small effect size (ES = 0.29) and standardized response mean (SRM = 0.44).

**Conclusion:**

The FRI-Ar was developed, and it showed good reliability and validity. However, it revealed the responsiveness with the small change. It can evaluate both pain and functional limitations in acute and subacute LBP patients. Before using it in the Arabic population with acute and subacute LBP, it is recommended to conduct further research to test internal and external responsiveness using an external criterion with a more extended follow-up period and suitable interventions.

## 1. Introduction

Low back pain (LBP) is one of the most common causes of disability and can lead to significant adverse outcomes from a social, psychological, and economic perspective. It is the most common musculoskeletal disorder requiring healthcare interventions [1]. It would affect more than 80% of the population at some point in their lifetime [2]. It ranks 6^th^ on the global burden of diseases, with 40%–60% of individuals with acute LBP still reporting pain after a year and 5%–7% with chronic pain [3]. Concerning the prevalence of LBP among adults, higher rates were found in high-income countries (30%) than in low-income countries (18.2%), with a higher prevalence in females than in males [4]. In Saudi Arabia, the prevalence of LBP is 18.8% among the general population [5] that becomes a significant public health issue [6].

Besides, previous literature identified LBP as a significant public health concern and a substantial problem for healthcare services [7, 8]. It is observed that functional disability is one of the significant factors that affect patients with LBP [2, 3], which is associated with increased healthcare seeking [4]. It is a risk factor for chronic pain that contributes most to long-term disability, morbidity, and healthcare and societal costs [9]. Patient-Reported Outcome Measures (PROMs) can evaluate a patient's functioning status regarding activity limitations and participation restrictions (reflecting the current World Health Organization disability classification criteria) [10]. The measurement of activity restrictions and the ability to perform daily tasks are crucial in determining the functional skill capability in patients with LBP. Furthermore, the data collected through PROMs are potentially more beneficial than traditional approaches, such as range of motion or muscle power [11]. Among the healthcare professionals (HCPs), physiotherapists (PTs) widely use several self-report outcome measures (i.e., questionnaires) for measuring a patient's health status or treatment outcomes. Specifically, patient-reported outcome tools to assess LBP consist of the Numerical Pain Rating Scale (NPRS), the Visual Analogue Scale (VAS) for pain intensity, the Roland-Morris Disability Questionnaire (RMDQ), the Oswestry Disability Index (ODI) for functional status, and the Short Form 36 Health Survey (SF-36) for general health status [12]. Likewise, the Functional Rating Index (FRI) is also a self-report scale used to assess pain and function to determine a patient's level of disability [13]. It was developed based on two earlier questionnaires: the ODQ [14] and the Neck Disability Index [15]. It has been widely used to evaluate LBP in several populations in whom it has demonstrated satisfactory reliability, validity, and responsiveness [16, 17].

Moreover, several outcome measure scales assessing the functional status of LBP are primarily available in the English language. This condition poses a significant challenge for both non-English speaking clinicians and the patients since the language is a significant barrier that impacts their access and quality of healthcare. One solution to successfully fill such a gap in healthcare services for non-English speakers is the provision of non-English health educational materials [18]. Hence, the provision of outcome measures in Arabic would be helpful for the non-English speaking population in Arab countries. Researchers in such non-English-speaking countries could create their PROMs or modify current English questionnaires through translation to overcome this issue. Adapting existing English language questionnaires into Arabic through translation is simpler, more efficient, and less time-consuming [19]. In such a condition, it is paramount to conduct the process of cross-cultural adaptation, which involves translators, HCPs who typically use the questionnaire, and researchers who understand clinimetrics. It includes initial translation, synthesis, back translation, expert committee review, pilot testing of the draft translation, and psychometric evaluation [20]. Most questionnaires used by the PTs in other languages were originally developed in the English language. The cross-cultural adaptation of those questionnaires would enable comparisons of different populations and permit the exchange of information across cultural and linguistic barriers.

Like other self-report outcome measures, the FRI was originally developed in English for LBP and neck pain (NP) [13]. Due to its clinical usefulness, simplicity, and excellent psychometric properties [21], the FRI has been cross-culturally adapted into eight languages: Turkish [15], Korean [22], Brazilian-Portuguese [23], Persian [24], Chinese [16], Thai [25], Spanish [26], and Italian [27]. However, there is no Arabic version of FRI to evaluate the functional status of individuals suffering from LBP. The development of such an Arabic version of FRI (FRI-Ar) would be helpful for both HCPs and patients. Hence, this study intended to adapt the FRI into the Arabic language cross-culturally and to analyze the psychometric properties of the FRI-Ar in cases with acute/subacute LBP.

## 2. Materials and Methods

### 2.1. Methods

This study was conducted in two phases. In the first phase, the English version of the FRI was cross-culturally adapted to the FRI-Ar as per the guidelines for cross-cultural adaptations of assessment tools [28]. In the second phase, the clinometric properties of the FRI-Ar were analyzed, including reliability, validity, internal consistency, and responsiveness.

### 2.2. Questionnaires

The questionnaires used in this study include the Arabic version of FRI, RMDQ, ODI, NPRS, Patient-Specific Functional Scale (PSFS), and Global Perceived Effect Scale (GPE). All these questionnaires were directed by the availability, recognized psychometric properties, and nonsuperiority of other questionnaires. The description for these questionnaires is as follows:

#### 2.2.1. Functional Rating Index

**Figure 1:**
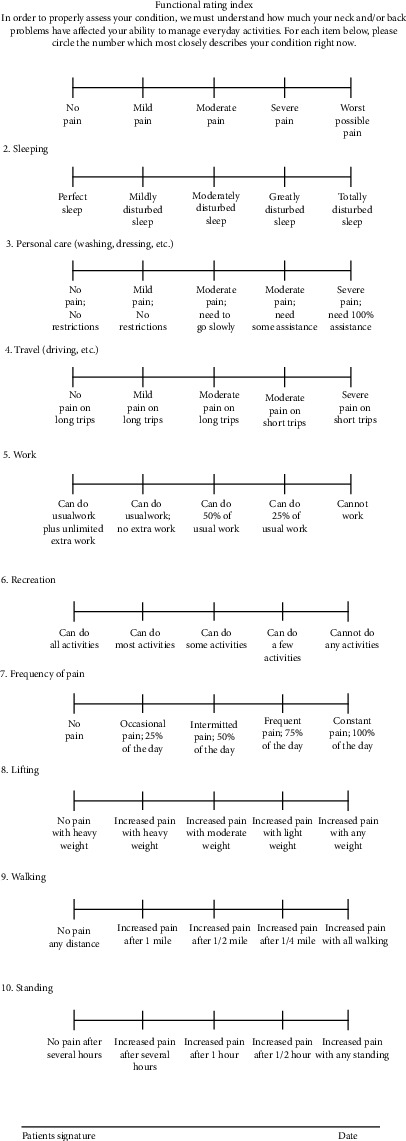
Functional Rating Index-English (FRI-En) questionnaire.

**Figure 2:**
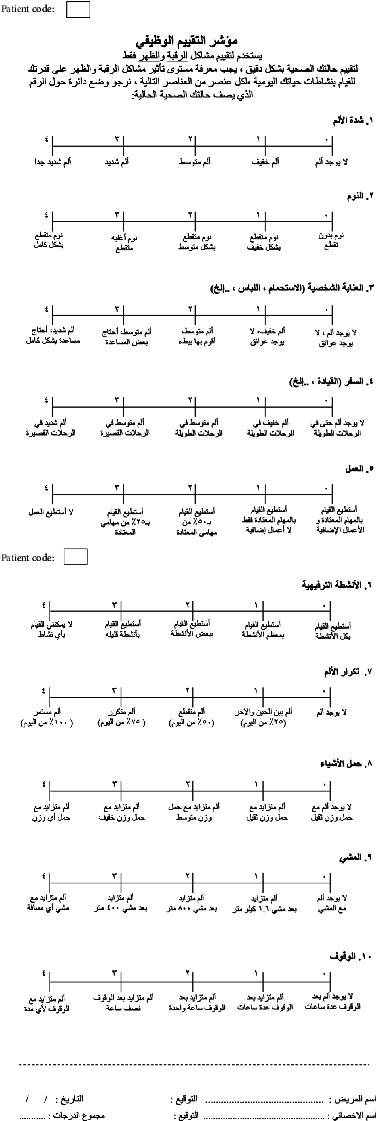
Functional Rating Index-Arabic (FRI-Ar) questionnaire.

The original English version of the FRI includes ten items intended to measure spinal musculoskeletal function and pain as perceived by the patients. The ten items constituting the FRI are pain intensity, sleep, personal care, travel, work, leisure activities, pain frequency, lifting, walking, and standing to gain insight into the functional status. Eight of these ten items are related to everyday activities that could be impaired by a spinal condition, whereas the remaining two are related to pain characteristics. Respondents must indicate their capacity to undertake an activity and/or the current level of pain experienced by awarding a score of 0 to 4, with 0 denoting no pain or complete functionality and the score “4” indicating maximum pain and/or complete impairment [13, 21] (see Figure 1 (FRI-English version)). Like the original version, the FRI-Ar version (see Figure 2 (FRI-Arabic version)) includes ten items focusing on assessing spinal musculoskeletal function and pain as perceived by the patients. These items are pain intensity, sleep, personal care, travel, work, leisure activities, pain frequency, lifting, walking, and standing. Among these ten items, eight are related to everyday activities that could be impaired by a spinal condition, whereas the remaining two are related to pain characteristics. This questionnaire adapted the same options (i.e., 0 to 4) used in the original version. The respondents can report their ability to perform an activity and/or their current pain level.

#### 2.2.2. Roland-Morris Disability Questionnaire

The RMDQ is based on 24 items from the Sickness Impact Profile (SIP) related to various facets of everyday life, most pertinent to LBP and including the added phrase “because of my back pain.” The scale comprises 24 items with two responses for each item, namely, “yes” and “no.” Therefore, the total score of that scale ranged between 0 and 24, with 0 denoting lack of impairment and a score of 24 indicating significant impairment [29]. The Arabic version of the RMDQ-24 has been confirmed valid and reliable [30].

#### 2.2.3. Oswestry Disability Index

The ODI is intended to shed light on the current functional status of patients. There are six statements per item, which must be rated using a scale ranging from 0 to 5. The highest possible score is 50, and this value is doubled to obtain a percentage. Functional status is subjectively classified into five categories: minimal (0%–20%), moderate (21%–40%), serious (40%–60%), crippled (61%–80%), and complete impairment (81%–100%) [31]. A previous study has used the Arabic version of ODI [32].

#### 2.2.4. Patient-Specific Functional Scale

In PSFS, the participants were required to list three activities that they found challenging or impossible to perform due to their back pain. Those activities had to be rated on an eleven-point scale from 0 (inability to undertake activity) to 10 (complete ability to undertake activity at the same level as prior to the back pain) [33, 34]. During follow-up, the participants were given their initial scores and were asked to provide a new score for every activity based on their perception of ability at present.

#### 2.2.5. Numerical Pain Rating Scale

The 11-point NPRS was used to determine the average intensity of pain that the participants felt over a day, with 0 denoting lack of pain and 10 indicating maximum pain [35]. The NPRS Arabic version has already been validated and proven reliable and comparable to the English version (Alghadir et al. [36]).

#### 2.2.6. Global Perceived Effect Scale

An Arabic version of the eleven-item Likert-type Global Perceived Effect Scale (GPE) was employed to gain insight into participants' perceptions of physiotherapy outcomes. This scale can be used as an external criterion to modify the clinical significance. There are three possible responses, namely, 5 (full recovery), 0 (no effect), and -5 (marked worsening) [37].

### 2.3. Translation and Cross-Cultural Adaptation

**Figure 3:**
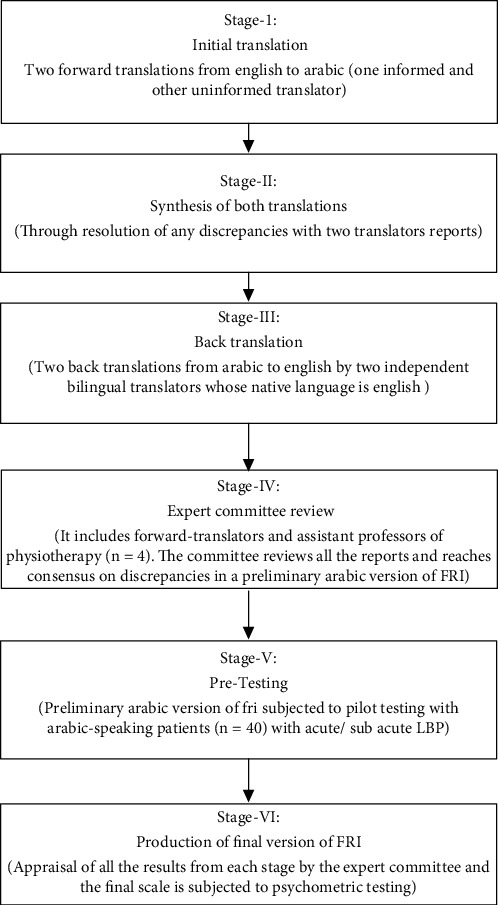
Stages of translation and cross-cultural adoption of Functional Rating Scale-Arabic (FRI-Ar).

The procedure adopted to carry out the cross-cultural adaptations of FRI-Ar includes initial translation, synthesis, back translation, expert committee review, pilot testing of the draft translation, and psychometric evaluation (Figure 3). First, two independent bilingual translators whose mother tongue was Arabic prepared the Arabic translation from the original English version of the FRI. One translator was a physiotherapist and was aware of the concept of this study. The second was a professional translator who was unaware of the study but familiar with the cultural and linguistic peculiarities of the Arabic language. Upon initial translation, both translators met to compare their translations and mutually addressed the differences and inconsistencies to generate a single prefinal FRI-Ar version. Following this, a back translation from the prefinal FRI-Ar version into English was carried out by two other independent bilingual (English and Arabic) translators, whose native language is English and were uninformed about the original English version. As a next step, an expert committee consisting of forward translators and assistant professors of physiotherapy (*N* = 4) reviews the initial English language questionnaire, the FRI-Ar version, the consensus Arabic translation, the English back translations, and all the related notes taken during the process. This expert committee finalized a preliminary Arabic version of FRI equivalent to the original FRI questionnaire. Then, the preliminary Arabic version of FRI was subjected to pilot testing with forty Arabic-speaking patients with acute/subacute LBP. Those patients were asked to complete the prefinal FRI-Ar version. This pretesting helps evaluate the questionnaire's comprehensibility and provides final input on its language. Lastly, the pilot tested FRI-Ar was subjected to psychometric testing.

### 2.4. Testing of Psychometric Properties of FRI-Ar

The population of this study constituted all patients with acute/subacute LBP visiting a private physical therapy center in Dammam, Kingdom of Saudi Arabia, between August 2020 and November 2020. Using a criterion-based sampling approach, 200 patients who met specific criteria were recruited in which the inclusion criteria consisted of patients of both genders, who are fluent in Arabic, between 18 and 80 years with the presence of acute LBP (<12 weeks), and with or without referred leg pain. The patients who have suspected pathological spine disorders were excluded, namely, fractures, spinal infections or malignancy, ankylosing spondylitis, rheumatoid arthritis, or other inflammatory diseases and psychiatric disorders. The ethical approval for this study was obtained from the Institutional Review Board, Deanship of Scientific Research, Imam Abdulrahman Bin Faisal University, Dammam, Saudi Arabia (IRB-PGS-2021-03-016). The participation of patients was voluntary, and they were given a detailed written and verbal explanation of the study's rationale, procedures, and methods. All those participants signed a consent form, and the collected data were kept confidential and stored electronically under the custody of the researchers.

### 2.5. Procedure

In this study, two Arabic-speaking physiotherapists working in the study setting volunteered to help the researchers during the data collection. Those PTs were explained with the research objectives, procedures, and outcome measures. Further, those PTs received formal training regarding the standard data collection protocol and were instructed to obtain informed consent from the patients.

All patients (*N* = 200) who met the inclusive criteria were administered with FRI-Ar and other Arabic questionnaires such as RMDQ, ODI, PSFS, NPRS, and GPE at baseline during the first visit.  Table 1 summarizes the psychometric properties of the Arabic version of other questionnaires used in this study. All those patients (*N* = 200) were subjected to the baseline measurement during the first session. Following this, 120 patients who completed the questionnaires were included for subsequent measurement 24 hours after the baseline (test-retest reliability) (second session). In contrast, the remaining 80 patients were excluded because of incomplete questionnaires and unwillingness to participate in follow-ups (i.e., 24 hours and two weeks). Furthermore, 120 patients completed the questionnaires after two weeks of treatment (test responsiveness) (third session) with no dropouts. The time intervals (24 hours for reproducibility and two weeks for responsiveness) were selected based on the review of the prognosis of acute LBP, which stated that LBP patients often improve quickly [38] but seem to detect improvements in 2 weeks but not in 24 hours. Also, it was considered unlikely that a patient's condition would change substantially within 24 hours, and therefore, this period was chosen [39]. After collecting the participants' answers, the authors reviewed them to ensure satisfactory completion and filed the records. The identity of the participants was not collected, and a specific code was allocated to every participant to ensure complete anonymity throughout the study.

### 2.6. Data Analyses

All data were statistically analyzed using the Statistical Package for the Social Science SPSS program (version 23) with a *p* ≤ 0.05. The Shapiro-Wilk test was used to screen the data distribution, and the variables were assessed for normality. Mean and standard deviations (SD) were calculated at the item level, and total scores for both administrations (at 24 hours and two weeks) of the FRI-Ar were obtained.

#### 2.6.1. Reliability

Internal consistency of the FRI-Ar was evaluated by calculating Cronbach's *α* at baseline. The item-scale correlation coefficients between single items and the total scores of the scale were evaluated. These values > 0.8 are considered to have good consistency [23]. Test-retest reliability was assessed using the intraclass correlation coefficients (ICC). The primary measure of reliability was based on an the ICC_(2.1)_ model with a two-way random effects model of variance and an absolute agreement description of a single measure, as participants performed the FRI only once in each session. ICC values were interpreted as follows: <0.50, poor; 0.50–0.75, moderate; 0.75–0.90, good; and >0.90, excellent [40]. Furthermore, measurement error was examined by calculating the standard error of measurement (SEM). The minimal true change in the score for one person beyond measurement error was estimated by calculating the minimal detectable change at a 95% confidence level (MDC_95%_) [41]. The following formulas were used to calculate the SEM and MDC_95%_, respectively: $\text{SEM}=\text{SD}\times \sqrt{1-\text{ICC}}$ (SD is the standard deviation) [42] and $\text{MD}{\text{C}}_{95\%}=1.96\times \sqrt{2}\times \text{SEM}$ [40, 43]. Finally, the 95% limits of agreement (LOA) between the scores of the FRI-Ar on the baseline and the following administrations were visually assessed by constructing a Bland-Altman plot [44]. The records of only those patients classified as stable in sessions 2 and 3 were used to evaluate the reliability.

#### 2.6.2. Validity

Construct validity was assessed by correlating the FRI-Ar with the other Arabic questionnaires (RMDQ, ODI, PSFS, NPRS, and GPE) using the Pearson correlation test (*r*). The correlation coefficient (*r*) values > 0.6 are considered to be strong [23, 43].

#### 2.6.3. Sensitivity

Responsiveness estimates how well a questionnaire detects changes over time or changes due to treatment [45]. Sensitivity to change, or responsiveness, of the FRI-Ar, NPRS, GPE, RMDQ, ODI, and PSFS was measured using the effect size (ES) and standardized response mean (SRM). The effect size (ES) is calculated as the mean difference between the scores at baseline and follow-up, divided by the standard deviation of the score at baseline [46]. The SRM is calculated as the mean score difference between baseline and follow-up, divided by the standard deviation of the change [47]. An ES of 0.20, 0.50, and 0.80 or greater indicates a small, moderate, and large change, respectively [48]. According to Chansirinukor et al. [49], the SRM value of 0.2 indicates a small change; 0.5, moderate; and ≥0.8, large change.

## 3. Results

### 3.1. Cross-Cultural Adaptation of the FRI-Ar

Minor changes were carried out during the translation phase to specific terms where a direct translation into the Arabic language is challenging. For instance, item 9 (walking) in the FRI-English version utilized the unit “mile,” i.e., 1/4 mile, 1/2 mile, and 1 mile, which is rarely used by the Arabic-speaking population. Hence, the translators opted for the unit “meter” for that item, i.e., 400 m, 800 m, and 1600 m. This change is also identified and utilized in the previous literature [50]. Further, the back translations and the original version of the FRI were compared, and no disparities were found. The expert committee and translators agreed with the adaptation recommended during the translation phase and the final Arabic version of the FRI. During the pilot testing, all items were answered and recognized satisfactorily, comprehensibly, and applicably by all 40 patients. Besides, no linguistic, cultural, or semantic difficulties were observed during the translation process of the other questionnaires such as RMDQ, ODI, PSFS, NPRS, and GPE.

### 3.2. Participant's Characteristics

A total of 200 patients with acute/subacute LBP were recruited in this study. The informed consent was obtained from all the participants before collecting the data. All the patients had completed the questionnaires at the baseline. Among those patients (*N* = 200), 120 completed the questionnaires and were included. Those (*n* = 80) who failed to complete the questionnaires were excluded at 24-hour retest. Subsequently, 120 patients completed the questionnaires at two weeks. The demographic and clinical characteristics of the participants at the baseline, 24 hours, and two weeks are summarized in Table 2.

### 3.3. Reliability

**Figure 4:**
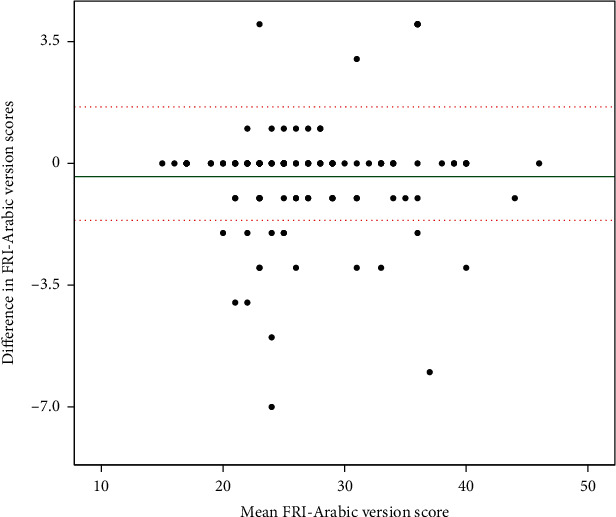
Agreement of the FRI-Ar scores between baseline and 24 hours using the Bland-Altman plot.

At the baseline, the internal consistency of the FRI-Ar was evaluated using the data of 200 patients. The overall Cronbach's alpha value of FRI-Ar was observed as 0.85 and rated as “good” [51, 52]. Subsequently, this study utilized the data of 120 patients at 24 hours from the baseline for measuring the reproducibility of FRI-Ar. Concerning the reliability, the items of the FRI-Ar instrument exhibited their reliability indexes varying from moderate (ICC_2,1_ 0.67; 95% confidence interval) to excellent (ICC_2,1_ 0.92; 95% confidence interval) reliability [43]. Further in total, the ICC_2,1_ value of the FRI-Ar instrument was observed as 0.85, which indicates the indicated substantial reliability [23, 43] (Table 3). Regarding the agreement, the SEM values of the items of FRI-Ar ranged from 0.12 to 0.21. The percentage of the SEM to the total score of FRI-Ar was observed as 2.9% (i.e., 1.17/40 × 100), which indicated that the instrument had a very good level of agreement [53]. The minimum detectable change (MDC) of 10 items of FRI-Ar was 0.33, 0.53, 0.36, 0.39, 0.53, 0.53, 0.39, 0.44, 0.58, and 0.55, respectively. The MDC for the total FRI-Ar was 3.24 (Table 3). Furthermore, the Bland-Altman plot assessed a 95% level of agreement (LOA) between the scores of FRI-Ar on the baseline and 24-hour retest. It showed a good agreement between FRI-Ar scores (Figure 4).

### 3.4. Construct Validity

Table 4 shows the correlation between all instruments at the baseline using the data from 200 patients. The FRI-Ar showed a significant moderate positive correlation with the RMDQ (*r* = 0.62), ODI (*r* = 0.65), and NPRS (*r* = 0.58). It showed a significant weak correlation with GPE (*r* = 0.27). The RMDQ showed a significant moderate positive correlation with ODI (0.58) and a weak correlation with NPRS (0.36). The ODI showed a significant moderate correlation with NPRS (0.52) and a weak correlation with GPE (0.36). The GPE showed a significant weak correlation with NPRS (0.36). Notably, PSFS showed a significant negative correlation with all other instruments (FRI-Ar, RMDQ, ODI, PSFS, and GPE) except NPRS.

### 3.5. Responsiveness of FRI-Ar

The responsiveness of the instruments was measured using the data collected from 120 patients at two weeks from the baseline. The results showed that the instruments varied with an effect size from small (RMDQ = 0.17; ODI = 0.17) to moderate (NPRS = 0.66). For FRI-Ar, ES was estimated as 0.29, which indicates a small change. The GPE was observed with an ES of 0.42, representing a small change. Notably, ES for RMDQ and ODI was observed as 0.17 and 0.17, respectively, which denotes a small change. For PSFS, ES was 0.56, indicating a moderate change. Likewise, the NPRS was found with an ES of 0.66, representing a moderate change. Besides, the SRM scores of the FRI-Ar were observed as 0.44, indicating a small change. Similarly, other instruments such as GPE, RMDQ, and ODI were observed with the SRM score between 0.34 and 0.49, demonstrating a small change. However, the SRM scores of NPRS and PSFS were observed as 0.71 and 0.65, respectively, indicating a moderate change. The results show that the FRI-Ar can measure the change in a patient's condition over time (Table 5).

## 4. Discussion

The current study was aimed at translating and cross-culturally adapting the FRI to Arabic and evaluating its clinimetric properties. Additionally, the instruments such as RMDQ, ODI, GPE, NPRS, and PSFS were used. While adapting the FRI cross-culturally, only item 9 of the original English version of FRI was slightly modified to make it more comprehensible among the Arabic-speaking population, and the remaining required no modifications. The FRI-Ar demonstrated Cronbach's alpha of 0.85, indicating the good reliability of the questionnaire. All items of the FRI measured the concept of the questionnaire. This outcome is similar to Cronbach's alpha of the Spanish version of FRI [26]. However, it is lower than Cronbach's alpha measured in the original English version of FRI (0.92) [13]. It is also in agreement with the other language versions, i.e., 0.86 for the Thai [25], 0.92 for the Brazilian-Portuguese [23], 0.96 for the Turkish [15], 0.90 for the Chinese [50], and 0.89 for the Persian [24].

Concerning the test-retest reliability, the ICC_2,1_ value for the items of FRI-Ar was observed between 0.67 and 0.92, representing moderate to excellent reliability. Overall, the FRI-Ar demonstrated substantial reliability with the ICC_2,1_ of 0.85. This finding is aligned with the previous versions, viz. Persian (ICC 0.81) [24], Chinese (ICC 0.95) [50], Thai (ICC 0.82) [25], Spanish (ICC 0.77) [27], and Brazilian-Portuguese (ICC 0.95) [23]. Furthermore, the agreement assessed by the percentage of the SEM to the total score was very good (2.9%) for FRI-Ar. This outcome is consistent with the earlier studies that demonstrated a very good level of agreement for FRI [25, 54]. Another measure of agreement is MDC_95%_, which reflects the minimal change in a score that could be inferred as actual change. A Thai version of FRI showed the MDC_95%_ of 2.5 for back pain patients [25]. However, FRI-Ar demonstrated the MDC_95%_ of 3.24, reflecting a change of 3 points from the baseline. This change is probably due to the actual change in the patients' functional disability status rather than measurement error. From these results, it is inferred that FRI-Ar is a reliable instrument.

While analyzing the construct validity, a moderate positive correlation was observed between the FRI-Ar and other Arabic versions of instruments (i.e., RMDQ, ODI, and NPRS). Similar findings were observed between the FRI and ODI [16], Spanish FRI and NPRS [26], and Thai FRI and Thai RMDQ (*r* = 0.68) [25]. Besides, FRI-Ar showed a weak positive correlation with the Arabic version of GPE and a negative correlation with the Arabic version of PSFS. However, an earlier study in LBP found that FRI was negatively correlated with GPE and PSFS [39]. Moreover, the presence of the hypothesized correlation between the FRI-Ar and the Arabic RMDQ indicates that both instruments measure similar constructs as these scales measured the pain and functional status of LBP patients. This finding is in line with the results of investigations using the original English version and the translated versions in patients with LBP [13, 15, 16, 23, 50, 55]. A previous study also observed high intercorrelations between the Pain Numerical Rating Scale (NRS) and the Brazilian-Portuguese FRI and RMDQ, which supported the construct validity of the FRI. The higher correlation between the FRI and pain NRS could be explained by 20% of total items of FRI being associated with the pain (i.e., the items 1 (pain intensity) and 7 (pain frequency)). In comparison, 4.2% of total items of RMDQ are associated with pain (i.e., item 13 (my back is painful almost all the time)) [23]. Another earlier study observed that FRI seems to be adequately valid as it showed a moderate correlation with ODI. However, FRI was extracted from ODI; hence, ODI might not be a perfect reference standard [16].

Besides, responsiveness refers to the ability of the instrument to detect changes over the course of intervention. It was calculated for the FRI-Ar, NPRS, GPE, ODI, PSFS, and RMDQ at baseline and two-week follow-up using SRM and ES. While reviewing the ES and SRM values, FRI-Ar, GPE, RIMDQ, and ODI showed a small change in the patient's condition. Such responsiveness might be observed since the patients with acute LBP are more likely to demonstrate actual changes than those with chronic LBP. Further, FRI-Ar, RMDQ, and ODI focused on pain and physical functioning. On the other hand, NPRS and PSFS demonstrated a moderate change in the patient's condition. These findings might be because NPRS only measures pain intensity, not physical functioning. The patients record their level of pain using NPRS. The PSFS only focuses on the specific activities that most trouble the patients, and the patients rate their function based on those specific activities [56]. In this study, PSFS dealt with three specific activities to which the patients provided their responses. Besides, the change in scores is likely due to the natural history of LBP and the effect of the physiotherapy intervention on the patient's condition.

Notably, the FRI-Ar presented the responsiveness with small ES (0.29) and SRM (0.44), which is better than the scores observed with the Arabic versions of RMDQ and ODI. Such a small change in FRI-Ar scores observed among 120 LBP patients might be due to a shorter period of follow-up (2 weeks from the baseline) and applied therapeutic interventions. Additionally, it may be easy and comfortable for the patients to record their actual change in symptoms using the FRI in their native language. In line with this finding, the Brazilian-Portuguese version of the FRI demonstrated the responsiveness with small ES (0.18), which was measured from 140 LBP patients following the four weeks of treatment. It is a reliable and valid tool for assessing disability in Brazilian-Portuguese-speaking LBP patients. It is appropriate for applying in clinical practice and research [23]. However, the current study is contrary to the results of Chansirinukor [57] who conducted the follow-up at two weeks and concluded the responsiveness of Thai FRI with large ES (1.34) and SRM (1.32) using 84 patients with LBP.

Moreover, previous studies reported the responsiveness of the original FRI in spinal cases. Their results were found with the ES of 1.24 [13], 0.64 [49], 2.08 (1 week), and 2.92 (4 weeks) [16]. Furthermore, few studies had observed the original FRI with an SRM of 0.70 [49] and the area under curve (AUC) of 0.93 [16]. The FRI is more reliable and responsive than the RMDQ-18. It consumes a shorter time to administer and complete and is used for LBP and neck pain patients. Further, it should be retested with functionally stable patients within one or two weeks of time interval [13, 49]. However, a previous study by Childs and Piva [16] found that the original FRI is less reliable but seems to have comparable validity and responsiveness, in which a follow-up was carried out at the end of the fourth week to measure responsiveness. On the other hand, the present study demonstrated the responsiveness of the FRI-Ar with the ES (0.29) and SRM (0.44) at two weeks from the baseline. Also, it showed that the FRI-Ar is a reliable and valid tool for assessing acute and subacute LBP patients. These differences in the values between the current study and earlier studies are mainly attributed to the various methodology adopted.

This is the first study to modify the FRI for an Arabic-speaking population and perform a psychometric examination of the FRI endpoints. The current study has presented psychometric evidence that the translation and culturally modified FRI-Ar are comparable to the English edition when utilized in an Arabic cohort of patients with acute or subacute LBP. However, the findings of this study are not easily generalized to the individuals with more focused symptoms or those who have undergone operative interventions. Moreover, factor configuration, ceiling, and floor effects and a modest clinically significant alteration in the FRI-Ar ranking necessitate further interpretation. Sample bias may also exist as the participants were not conscripted by a randomized method. Future studies `can be conducted using the FRI-Ar with the inclusion of varying LBP types and equal participation of females. This study has only revealed the internal responsiveness (ES and SRM); however, the external responsiveness of the FRI-Ar can be tested in upcoming studies using an external criterion of change such as GPE. Further research is warranted to obtain better responsiveness of this instrument by applying alternative therapeutic approaches or more extended follow-up periods.

## 5. Conclusion

The FRI-Ar showed good reliability and validity; however, it demonstrated responsiveness with small change. It would be an ideal self-reported outcome tool for clinical and scientific practice in Arabic patients suffering from acute or subacute LBP. It can be utilized to assess both pain and functional limitations in LBP patients. This Arabic version of the FRI is easy to conceive, quick to complete, and highly accepted and allows patients to grade limitations of activity and participation restrictions. However, it is recommended to conduct future studies to test internal and external responsiveness using an external criterion with a more extended follow-up period and appropriate therapeutic interventions before its general use among the Arabic-speaking population with acute and subacute LBP.

## Figures and Tables

**Table 1:** Psychometric properties of Arabic instruments used in this study.

Instruments	ODI	RMDQ	PSFS
Study	Algarni et al. [32]	Maki et al. [30]	Alnahdi et al. [33]
Internal consistency	0.886	0.729	0.75
Test-retest reliability	ICC = 0.99	ICC = 0.900	ICC = 0.86
Construct validity	*r* = 0.656^a^; *r* = 0.708^b^	*r* = 0.259^b^	—

^a^Correlated with the Roland-Morris Disability Questionnaire. ^b^Correlated with the Visual Analogue Scale. Abbreviations: *α*: Cronbach alpha: ICC: intraclass correlation coefficients; RMDQ: Roland-Morris Disability Questionnaire; ODI: Oswestry Disability Index; PSFS: Patient-Specific Functional Scale.

**Table 2:** Participant's characteristics at baseline and follow-ups.

Variables	Baseline (*n* = 200)	24 hours (*n* = 120)	2 weeks (*n* = 120)
Age (in years)^∗^			
18-29	71 (35.5%)	45 (37.5%)	45 (37.5%)
30-39	50 (25%)	28 (23.3%)	28 (23.3%)
40-49	47 (23.5%)	27 (22.5%)	27 (22.5%)
50-59	23 (11.5%)	12 (10%)	12 (10%)
60-69	9 (4.5%)	8 (6.7%)	8 (6.7%)
Gender^∗^			
Male	128 (64%)	79 (65.8%)	79 (65.8%)
Female	72 (36%)	41 (34.2%)	41 (34.2%)
BMI (kg/m^2^)^∗∗^	29.93 ± 5.66	30.52 ± 5.96	30.52 ± 5.96
Weight^∗∗^	81.34 ± 18.08	82.05 ± 18.22	82.05 ± 18.22
Height^∗∗^	164.47 ± 9.02	163.66 ± 8.29	163.66 ± 8.29
Marital status^∗^			
Single/other	48 (24%)	26 (21.7%)	26 (21.7%)
Married	152 (76%)	94 (78.5)	94 (78.5%)
Education^∗^			
Primary	3 (1.5%)	0 (0%)	0 (0%)
Intermediate	5 (2.5%)	5 (4.2%)	5 (4.2%)
Secondary	35 (17.5%)	17 (14.2%)	17 (14.2%)
Undergraduate	144 (72%)	89 (74.2%)	89 (74.2%)
Postgraduate	13 (6.5%)	9 (7.5%)	9 (7.5%)
Employment status^∗^			
Student	31 (15.5%)	19 (15.8%)	19 (15.8%)
Employee	129 (64.5%)	82 (68.3%)	82 (68.3%)
Not working	35 (17.5%)	16 (13.3%)	16 (13.3%)
Retired	5 (2.5%)	3 (2.5%)	3 (2.5%)
Duration of symptoms^∗^			
Acute LBP (from 1 day to <6 weeks)	67 (35.5%)	48 (40%)	48 (40%)
Subacute LBP (between 6 and 12 weeks)	133 (66.5%)	72 (60%)	72 (60%)
Instruments^∗∗^			
FRI-Ar (0-40)	26.22 ± 6.42	26.77 ± 6.58	25.27 ± 7.49
RMDQ (0-24)	6.4 ± 5.03	6.94 ± 5.21	6.03 ± 5.22
ODI (0-50)	23.93 ± 6.73	24.77 ± 6.54	23.64 ± 7.44
PSFS (0-30)	19.11 ± 4.59	18.16 ± 4.36	20.70 ± 4.75
NPRS (0-10)	6.87 ± 1.92	6.98 ± 1.93	5.85 ± 2.01
GPE (-5 to +5)	5.22 ± 2.19	5.52 ± 2.11	4.66 ± 2.20

^∗^Values expressed in frequency (*n*) and percentage (%). ^∗∗^Values expressed in mean ± standard deviation. Abbreviations: BMI: body mass index; SD (±): standard deviation; LBP: low back pain; NPRS: Numeric Pain Rating Scale; RMDQ: Roland-Morris Disability Questionnaire; ODI: Oswestry Disability Index; PSFS: Patient-Specific Functional Scale: GPE: Global Perceived Effect Scale.

**Table 3:** Reliability of the FRI-Ar.

FRI-Ar	ICC_2,1_ (95% CI)	SEM	MDC_95%_
Pain intensity	0.84 (0.78-0.89)	0.12	0.33
Sleeping	0.90 (0.86-0.93)	0.19	0.53
Personal care	0.86 (0.81-0.90)	0.13	0.36
Travel	0.67 (0.56-0.76)	0.14	0.39
Work	0.93 (0.91-0.95)	0.19	0.53
Recreation	0.86 (0.81-0.90)	0.19	0.53
Frequency of pain	0.92 (0.89-0.94)	0.14	0.39
Lifting	0.77 (0.69-0.84)	0.16	0.44
Walking	0.87 (0.82-0.91)	0.21	0.58
Standing	0.90 (0.86-0.93)	0.20	0.55
Total	0.85 (0.74-0.91)	1.17	3.24

Abbreviations: CI: confidence interval; ICC: intraclass correlation coefficient; FRI-Ar: Arabic version of Functional Rating Index; SEM: standard error of measurement.

**Table 4:** Pearson's correlation among the instruments at baseline.

Instruments	FRI-Ar	RMDQ	ODI	PSFS	GPE	NPRS
FRI-Ar	1	0.62^∗^	0.65^∗^	-0.35^∗^	0.27^∗^	0.58^∗^
RMDQ	0.62^∗^	1	0.58^∗^	-0.29^∗^	0.27^∗^	0.36^∗^
ODI	0.65^∗^	0.58^∗^	1	-0.19^∗^	0.36^∗∗^	0.52^∗^
PSFS	-0.35^∗^	-0.29^∗^	-0.19^∗^	1	-0.21^∗∗^	-0.14
GPE	0.27^∗^	0.27^∗^	0.36^∗^	-0.21^∗^	1	0.36^∗^
NPRS	0.58^∗^	0.36^∗^	0.52^∗^	-0.14	0.36^∗^	1

^∗^Significant at 0.001 level.

**Table 5:** Responsiveness of instruments.

Instruments	Baseline SD	Change in means	Change in SD	ES	SRM
FRI-Ar	6.42	1.88	4.26	0.29	0.44
NPRS	1.82	1.20	1.70	0.66	0.71
GPE	2.09	0.88	1.81	0.42	0.49
RMDQ	5.34	0.89	2.62	0.17	0.34
ODI	6.72	1.14	3.21	0.17	0.36
PSFS	4.46	2.51	3.86	0.56	0.65

## Data Availability

The dataset used is available from the corresponding author upon request.
